# Physical basis of amyloid fibril polymorphism

**DOI:** 10.1038/s41467-018-03164-5

**Published:** 2018-02-16

**Authors:** William Close, Matthias Neumann, Andreas Schmidt, Manuel Hora, Karthikeyan Annamalai, Matthias Schmidt, Bernd Reif, Volker Schmidt, Nikolaus Grigorieff, Marcus Fändrich

**Affiliations:** 1 Institute of Protein Biochemistry, Ulm University, 89081 Ulm, Germany; 2 Institute of Stochastics, Ulm University, 89081 Ulm, Germany; 3 Institute for Structural Biology, Helmholtz Zentrum München, 85764 Neuherberg, Germany; 4 Munich Center for Integrated Protein Science (CIPS-M) at Department Chemie, Technische Universität München (TUM), Lichtenbergstr. 4, 85747 Garching, Germany; 5 Janelia Research Campus, Howard Hughes Medical Institute, 19700 Helix Drive, Ashburn, VA 20147 USA

## Abstract

Polymorphism is a key feature of amyloid fibril structures but it remains challenging to explain these variations for a particular sample. Here, we report electron cryomicroscopy-based reconstructions from different fibril morphologies formed by a peptide fragment from an amyloidogenic immunoglobulin light chain. The observed fibril morphologies vary in the number and cross-sectional arrangement of a structurally conserved building block. A comparison with the theoretically possible constellations reveals the experimentally observed spectrum of fibril morphologies to be governed by opposing sets of forces that primarily arise from the β-sheet twist, as well as peptide–peptide interactions within the fibril cross-section. Our results provide a framework for rationalizing and predicting the structure and polymorphism of cross-β fibrils, and suggest that a small number of physical parameters control the observed fibril architectures.

## Abstract (web summary)

Amyloid fibril structures can display polymorphism. Here the authors reveal the cryo-EM structures of several different fibril morphologies of a peptide derived from an amyloidogenic immunoglobulin light chain and present a mathematical analysis of physical factors that influence fibril polymorphism.

## Introduction

Amyloid fibrils are filamentous protein states that were originally identified in the context of systemic amyloidosis or neurodegenerative diseases such as Alzheimer’s and Parkinson’s^1-3^. More recently, however, amyloid-like fibrils have been suggested as novel devices in materials sciences, chemical engineering, and bionanotechnology^4^. Amyloid fibrils possess a unique structure that is characterized by a cross-β-sheet conformation in which β-strands run transversely to the main fiber axis and form an intermolecular network of hydrogen bonds^1,5^. They consist of one or multiple protofilaments (PFs)^6–11^. They usually exhibit a width of 5–20 nm^6,7,11–13^, an overall polar topology^1,6,8–11,14^, a left-handed fibril supertwist^6–9,11,14^, and a twofold helical symmetry^6,8,9,11,14^. A particular remarkable feature about amyloid fibril structures is their polymorphism.

Polymorphism refers to structural variations among different amyloid fibrils formed by a particular polypeptide chain, which can usually be observed with single-particle techniques when comparing the individual fibril structures in a sample^6,7,11–13,15^. This intrasample polymorphism can be found irrespective of whether fibrils were formed in vitro^6,7,11,15^ or extracted from a patient or diseased animal^12,13,16^. Changing the conditions of fibril formation affects the spectrum of fibril morphologies that can be obtained within a test tube^15,17^, and different fibril morphologies may underlie the formation of different variants of amyloid diseases in vivo. Evidence in this regard has been provided for the aggregates formed by Aβ peptide, prion protein, transthyretin, or serum amyloid A protein^18–21^.

Due to this polymorphism, predicting the formation of amyloid structures can be considered to be more complex than the conventional protein-folding problem, as the protein-folding reaction connects a given amino acid sequence with an essentially singular globular conformation^22^. In contrast, solutions to the “protein misfolding problem” are required to explain the polymorphic variations and the formation of multiple conformational end states by a polypeptide chain^6,7,11–13,15,17,23^. Previous analyses of polymorphic fibrils have revealed them to be linked with alterations in the number, orientation, or substructure of the PFs constructing different fibril polymorphs^6,7,11,16,23,24^ and have led to hypotheses concerning the possible modes of alternative packing of polypeptide chains^25^. However, it remains challenging to explain the specific structural assemblies seen within an experimental sample.

Using electron cryomicroscopy (cryo-EM), we have analyzed the polymorphism of fibrils formed by a peptide fragment from an immunoglobulin light chain causing systemic AL amyloidosis^8^. This peptide, termed AL1, possesses a sequence (IGSNVVTWYQQL) that is devoid of charged amino acid side chains, confining all of its ionic groups to the peptide N- and C-termini. For one of its fibril morphologies, termed hereafter morphology I, we previously obtained a cryo-EM reconstruction at sub-nm resolution^8^. This fibril encompassed six PFs, each consisting of a face-to-face packed pair of two cross-β sheets. The fibril exhibited only two ways by which two PFs were packed within the mature fibril. One possibility involved a self-complementary packing of the lateral surfaces of two β-sheets that is mediated by inter-PF contacts between the hydrophobic and polar amino acid side chains. The other possibility involved electrostatic contacts between the complementary charged peptide N- and C-termini at the edges of the β-sheets in adjacent PFs. In this study, we extended this analysis to nine additional fibril morphologies formed by the AL1 peptide under the same conditions as morphology I. The obtained cryo-EM reconstructions were used to develop an algorithm to rationalize and predict the formation of specific fibril morphologies from this peptide.

## Results

### Cryo-EM reconstructions of different fibril morphologies

**Fig. 1:**
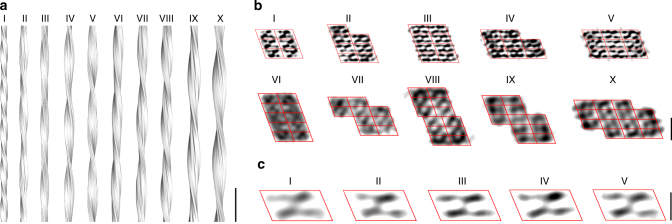
Structural polymorphism of AL1 peptide fibrils. **a** Side view of reconstructions of fibril morphologies I–X (scale bar, 50 nm). **b** Cross-sectional slices of the reconstructed densities superimposed with a lattice of parallelograms (red). Reconstructions of morphologies I–V were filtered to 10 Å and morphologies VI–X to a resolution corresponding to their FSC values at 0.143 (scale bar, 5 nm). **c** Aligned and averaged parallelograms of morphologies I–V sharing a quasi-twofold symmetry. The data from morphology I were included as published^8^ (scale bar, 2 nm)

Analysis of the width of AL1 peptide fibrils with cryo-EM revealed a broad range of diameters from 8 to 22 nm (Supplementary Figure 1), demonstrating the variation of fibril structures in this sample, which we previously used to identify morphology I^8^. We now obtained reconstructions from nine additional fibril morphologies (II to X) that are present in this sample. For four reconstructions, we achieved resolutions of better than 1 nm (morphologies II to V) when estimated using the Fourier shell correlation (FSC) criterion at 0.143 (Supplementary Figure 2 and 3, Supplementary Table 1). The remaining fibrils were reconstructed at resolutions of 1.3–2.0 nm (Supplementary Table 1). Each fibril morphology presented a unique cross-sectional architecture (Fig. 1b). The reconstructions of morphologies III, V, VI, and VIII–X remained essentially unaltered when twofold axial symmetry was imposed during refinement and reconstruction (Supplementary Figure 4), suggesting that this symmetry was present. In contrast, the reconstructions of morphologies II, IV, and VII were strongly altered when twofold axial symmetry was imposed, indicating that these filaments do not possess this symmetry (Supplementary Figure 4), and morphologies II, IV, and VII are shown here without an imposed symmetry. All fibrils were identified as polar by the image-processing software FREALIX^26^. We imposed a left-handed helical twist during reconstruction consistent with platinum side shadowing^8^. Each fibril morphology presented a unique cross-sectional architecture (Fig. 1b) from which we could calculate the polar moment of inertia *I* as a geometrical characteristic (Supplementary Methods). *I* represents a measure of the resistance of a fibril toward twisting and correlates, in the set of analyzed fibrils, with the pitch (Supplementary Figure 5). This finding relates to previous observations made with various amyloidogenic polypeptide chains, in which the pitch correlates with either *I* or fibril width^6,11,15,27,28^. It indicates that the torsional features of the global fibril morphology, and thus the three-dimensional (3D) fibril structure, are determined by the two-dimensional (2D) architecture of the fibril cross section.

### A conserved building block constructs the different fibrils

Analysis of the cross-sections of the various fibril morphologies identified a common building block that could be outlined by a parallelogram (Fig. 1b). A cross-correlation analysis of morphologies I–V established the average dimensions of axes *a* and *b* of the parallelogram at 4.22 ± 0.31 nm and 2.29 ± 0.09 nm. The angle γ between the two axes was determined at 69.8 ± 3.2° (Supplementary Table 2). As each of these building blocks corresponded to the cross-section through a PF, the analyzed fibrils consisted of six to fourteen PFs, one of the highest PF numbers reported so far. The analyzed fibrils could be grouped into a “family tree” according to their increase in complexity (Supplementary Figure 6). Although finer details of the peptide conformation, such as the specific backbone dihedral angles or side chains, were not resolved at the current resolutions, each parallelogram enclosed two elongated density regions at a spacing distance of ~11 Å. These densities were observed within fibril morphologies I–V (Fig. 1) and with lower confidence also within fibril morphologies VIII, IX, and X (Supplementary Figure 7). Their size, density modulation, and quasi-twofold symmetry implied a face-to-face packed peptide dimer to underlie the fibril building blocks^8^.

### Conservation of the interactions between the peptide dimers

There were primarily two ways by which two peptide dimers (or parallelograms) could be packed within a fibril cross-section. One possibility involved an in-register packing along axis *a* of the parallelogram (long side interactions, LSIs) and the other, also in-register, along axis *b* (short side interactions, SSIs). In-register LSIs and SSIs were shared by all 10 fibril morphologies, although fibril morphology VIII showed an additional off-register LSI in the fibril center (Supplementary Figure 7). LSIs were previously shown to arise from nonionic interactions between the exclusively hydrophobic and polar amino acid side chains, while SSIs involved electrostatic contacts between adjacent peptide N- and C-termini^8^. The number of LSIs per fibril tended to be greater than the number of SSIs. Some fibril morphologies show discernible gaps in the corners of their cross-section, corresponding to the size of a parallelogram. The parallelograms were only missing in the blunt corners of some of the cross-sections but never in the sharp corners, suggesting that unfilled sharp corners are structurally unfavorable.

### Analysis of the protomer structure by ss-NMR

**Fig. 2:**
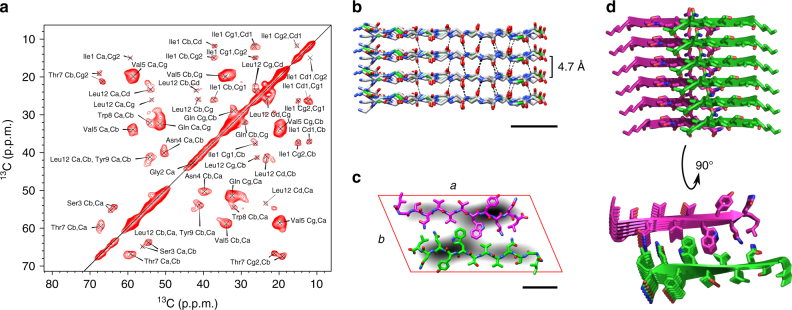
Protomer conformation revealed by ss-NMR. **a** The 2D ^13^C–^13^C proton-driven spin diffusion spectrum shows the cross-peaks between aliphatic carbon atoms. **b** Backbone model of the cross-β sheet implementing Φ/Ψ angles as predicted by the program TALOS+^29^ (scale bar, 1 nm). **c** Averaged parallelograms of morphologies I–V (gray) superimposed with a peptide dimer (green, magenta) as obtained by NMR. The placement of the peptides followed the considerations described previously for morphology I^8^ (scale bar, 1 nm). **d** Side view and top view of a six-layer stack illustrating the structure of a PF. Side-chain conformations were not determined by experiments and represent only conformations that are compatible with the packing shown here

**Fig. 3:**
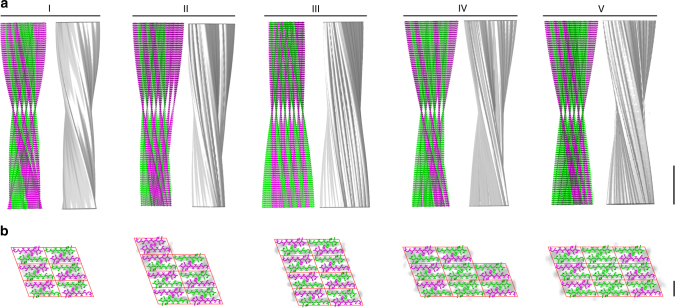
Peptide assembly of fibril morphologies I to V. **a** Side-by-side presentation of the experimentally obtained density and of the peptide assembly of fibril morphologies I to V (scale bar, 10 nm). **b** Peptide cross-sections showing an exposed N terminus (magenta) and those not exposed (green) within one peptide layer, superimposed with reconstructions (gray) and filtered to 10 Å. Side-chain conformations were not determined by experiments and represent only conformations that are compatible with the packing shown here. The 3D map of morphology I was included as published^8^ (scale bar, 2 nm)

The structural conservation of the building blocks and that of the peptide conformation in different fibrils was supported by magic angle spinning (MAS) solid-state-NMR (ss-NMR) spectroscopy data obtained with uniformly ^13^C/^15^N-labeled AL1 peptide fibrils. Despite the clear polymorphism of the analyzed sample, the NMR measurements yielded well-resolved spectra (Fig. 2a) with methyl ^13^C resonances exhibiting a line width of ~130 Hz (0.33 p.p.m.). Most residues were associated with a single set of chemical shifts with the exception of Ser3 and Thr7, which resolved two sets of chemical shifts. However, the variations of these two residues did not discernibly affect the prediction of the peptide conformation by the program TALOS+^29^, which yielded essentially the same extended β-strand conformation with both sets of chemical shifts (Supplementary Figure 8c, d). The Φ/Ψ-pairs occurred at the right side of the diagonal of the Ramachandran plot (Supplementary Figure 8b), indicating a left-handed β-sheet twist when viewed in the direction of the backbone hydrogen bonds^30^. This orientation of the twist is consistent with the orientation of the fibril supertwist as established previously by platinum side shadowing^8^. A peptide model implementing the NMR-based Φ/Ψ constraints provided a good fit to the elongated densities enclosed by the parallelograms. Stacking multiple peptide dimers into a PF depicts a pair of two self-complementary cross-β sheets (Fig. 2b–d) and the structural hierarchy of fibril morphologies I–V (Fig. 3).

### Physical factors influencing fibril polymorphism

Having established that different fibril morphologies consisted of a common building block and that these fibrils show mainly two ways to pack the building blocks within the fibril cross-section, we wondered how many fibril morphologies (cross-sections) can be generated by a given number *n* of building blocks or PFs? Furthermore, why are only some observed by experiments? To illustrate this problem, we found 120 possible arrangements encompassing *n* = 6 building blocks with only one being observed by cryo-EM (Supplementary Figures 9, 10) and a total of 346,649 possible constellations for the entire range of 1 ≤ *n* ≤ 12 (Supplementary Figure 11), eight of which were observed. As a next step, we considered the energetic contributions *E*_l_, *E*_s_, and *E*_e_ provided by a LSI, SSI, and an empty sharp corner to the fibril stability and combined them to define an arbitrary energy score *E* for each fibril morphology as shown in Eq. (1) (for details please refer to the “Methods” section).

1 $$E = E_{\rm{l}}n_{\rm{l}} + E_{\rm{s}}n_{\rm{s}} + E_{\rm{e}}n_{\rm{e}} + \alpha I^2$$

**Fig. 4:**
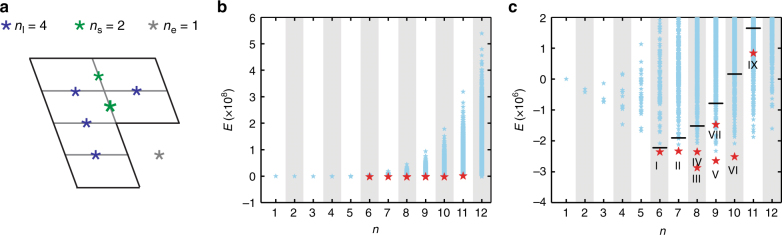
Mathematical analysis of the observed and theorized fibril morphologies. **a** Graphical representation of the definition of the parameters *n*_l_, *n*_s_, and *n*_e_ for a given fibril cross-section. **b** Plot of *E* for all theorized fibril morphologies or cross-sections (blue) vs. *n* as obtained by the even fit (gray columns). Hence, only white columns have predictive power for the even fit. Red symbols: experimentally observed fibril morphologies. **c** Close-up of **b**. Black horizontal division markers show the 1% cutoff of the most stable morphologies for each *n* value

In this equation, *n*_l_ refers to the number of in-register LSIs, *n*_s_ to the number of SSIs, and *n*_e_ to the number of empty sharp corners. Additionally, we considered the possible effects arising from *I*, the polar moment of inertia, which we scaled relative to *E* by the factor *α*. The definition of *E* followed the sign conventions of the Gibbs free energy (Δ*G*) in which a lower *E* value implies a higher thermodynamic stability. We then computed the values of *n*_l_, *n*_s_, *n*_e_, and *I* for all 346,649 fibril morphologies and split the resulting data set into two parts, one encompassing all fibril morphologies with an even number of PFs, and one encompassing all fibril morphologies with an odd PF number. Each subset was then evaluated with Eq. (1), termed hereafter as odd and even fits, to yield values for *E*_l_, *E*_s_, and *E*_e_ (Supplementary Table 4). These values were used to compute the stability of all fibril morphologies (Fig. 4, Supplementary Figure 12). This analysis possesses predictive power in those cases where the obtained values for *E*_l_, *E*_s_, and *E*_e_ were used to compute *E* of fibrils from the other subset; that is, when odd-fit data were used to predict even-numbered fibrils and vice versa.

On the basis of this approach, we found that the experimentally observed fibril morphologies belong to the 1% most stable cross-sectional arrangements for each respective *n* value (Fig. 4c, Supplementary Figure 12b). Morphologies I, II, III, IV, V, and VI represented the most stable fibril architectures for *n* = 6, 7, 8, 9, and 10. Together with fibril morphology VII, they ranked within the 67 (even fit) or 49 (odd fit) most favorable fibril morphologies overall (Fig. 4, Supplementary Figure 12). There is an intermediate region of *n* in which the formed fibrils could become particularly stable. This intermediate region was observed irrespective of whether we analyzed the lowest energy state at each *n* value (Supplementary Figure 13a, b) or whether we counted the number of fibril morphologies below a certain threshold of *E* (Supplementary Figure 13c, d). The intermediate region coincided with the population maximum when analyzing the fibril ensemble with mass-per-length (MPL) measurements (Supplementary Figure 13e).

The signs of E_s_ and E_l_ were found to be negative based on the fit and of E_e_ to be positive. In other words, the fibril structures arose from opposing sets of the following forces: stabilizing forces associated with LSIs and SSIs, and unfavorable forces originating from an unfilled sharp corner and *I*. The compromise between these opposing sets of forces was fibrils with relatively compact cross-sections (low *I* values, high n_l_, and n_s_ values) (Supplementary Figure 12c), which is in accordance with the tightly knit nature of the fibril cross-sections seen by cryo-EM (Fig. 1). It is a consequence of our theory that the fibril width adopts a relatively narrow range of values, which we found by cryo-EM to range from 6 to 22 nm in AL1 peptide fibrils (Supplementary Figure 1). The presently developed theory might apply to other cross-β-fibril systems as indicated in Supplementary Figure 14 for the fibrils formed from a peptide fragment of transthyretin (TTR)11.

## Discussion

In this study, we have used cryo-EM to characterize the structural variations of AL1 peptide fibril morphologies. We found that different fibril morphologies arise from different combinations of similarly structured building blocks. The obtained cryo-EM data enabled us to analyze the formation of the observed fibril structural variants as compared to the theoretically possible alternative fibril arrangements. We found that a small number of physical parameters controls fibril architectural features in the experimental sample.

The simplifications applied in our analysis give rise to a number of limitations explaining the discrepancies between our theory and some sample properties. For example, we have reconstructed only ten fibril morphologies by cryo-EM, but additional fibril morphologies were identified in our cryo-EM images. Thus, other fibril configurations beyond the ones reconstructed here could be theorized to have a similar stability. Our analysis was further restricted to consider only in-register LSIs and SSIs, whereas morphology VIII encompasses additional off-register LSIs within the fibril core. In addition, the analyzed fibril morphologies were formed under a single set of conditions, suggesting that the AL1 peptide may be able to adopt fibril morphologies beyond the ones considered by our theory.

We ignored the peptide–peptide interactions along the main fibril axis and assumed *E*_l_, *E*_s_, and *E*_e_ to be independent from their exact radial and azimuthal positions in the twisted fibril helix. This assumption may break down progressively as the fibril width increases, limiting the confidence of our analysis at larger *n* values. Equation (1) is also solely based on energy and does not consider kinetic contributions in defining the polymorphism of fibril morphologies^31^. As kinetic factors can be assumed to favor fibrils with a small number of PFs, which may nucleate and grow out faster than fibrils consisting of many more PFs, our analysis likely overestimates the formation of fibrils with large *n* values.

Other limitations come from the fact that we cannot measure the stability of single morphologies experimentally nor derive a theoretical energy threshold to discriminate between possible and impossible fibril morphologies. Hence, it is difficult to compare our *E* score with Δ*G* values obtained for specific fibril morphologies. However, we have noted that fibril morphologies VII and IX, which were rare in our sample and contributed only a few raw fibril images to our reconstructions (Supplementary Table 1), presented a low stability by our *E* score. In contrast, morphology I did not result in being the most stable fibril morphology in both the odd and even fit, even though it was the most abundant morphology in solution. Ultimately, our analysis depends on the knowledge of the PF structure, which is difficult to predict for longer polypeptide chains, such as Aβ peptide, α-synuclein, or tau, which encompass non-β segments or regions of conformational variability^1,3,9,16,32^.

Despite these limitations, our theory is able to explain the formation of the majority of the AL1 peptide fibrils observed by experiments. This ability implies that the incorporated physical properties are relevant for the formation of these cross-β fibrils. We identified two opposing sets of forces to control the structure and formation of AL1 peptide fibrils, favorable forces arising from LSIs and SSIs, and unfavorable forces arising from unfilled sharp corners and *I*. At the molecular chemical level, these findings imply that the desire of the peptide to make intermolecular interactions within the cross-sectional plane, which promotes the lateral fibril growth and the formation of thick fibrils, is in competition with the twisting of the cross-β-sheet structure. As the β-sheet twist is counteracted by *I*, increasing the lateral extension of the fibril cross-section and the fibril thickness will make the formation of fibrils progressively unfavorable.

It is clear that several of the specific structural features considered by our quantitative analysis with Equation 1, such as the existence of LSIs and SSIs as well as empty sharp corners, may not exist in this form in other amyloid systems and specifically in those with a more complex fold of the polypeptide chain in the fibril. However, our theory incorporates a number of general structural features of amyloid fibrils. For example, a twisted fibril structure has been described for the amyloid fibrils formed from many polypeptide chains^6–9,11,14,16,24^, and there is, for many amyloid systems, evidence for a correlation between the fibril pitch and the fibril width or *I*^6,11,15,27,28^. Moreover, the lateral fibril width of amyloid fibrils ranges frequently between 5 and 20 nm^6,7,11–13,16,32^, similar to AL1 peptide fibrils (Supplementary Figure 1), and the formation of peptide–peptide interactions orthogonal to the direction of the backbone hydrogen bonds has been crucial in defining the steric zipper structures of amyloid fibril spines^1,25^. Moreover, Supplementary Figure 14 demonstrates that our theory can also be applied to rationalize the fibril structures formed from a peptide fragment of TTR^11^. Hence, it appears that the presently described theory of a competition between cross-sectional peptide–peptide interactions and fibril twist could be relevant for amyloid structures more generally.

Our observations provide a framework toward predicting amyloid structures, which has to consider the following three complexity levels: (i) the fold of the polypeptide chain, (ii) the packing of one or several polypeptide chains into a PF, and (iii) the arrangement of multiple PFs into a fibril. While previous studies primarily focused on the first two complexity levels^33–36^, our study addresses the arrangement of multiple PFs into a fibril. This analysis was greatly enabled by the structural simplicity of AL1 peptide and the fact that the PF–PF contacts occurred almost exclusively via in-register LSIs and SSIs. Therefore, it will be a task for future studies to improve our theory to predict the possible PF–PF interactions of longer polypeptide chains, such as in Aβ and tau fibrils, for which cryo-EM has recently provided evidence for alternative arrangements of similarly structured PFs^6,16^.

## Methods

### Source of the peptide and conditions of fibril formation

Unlabeled and uniformly ^13^C/^15^N isotopically labeled variants of AL1 peptide were chemically synthesized at the Interdisziplinäres Zentrum für Klinische Forschung Leipzig, Core Unit Peptide-Technologien. Incubation of the peptide formed fibrils at a concentration of 5 mg/mL in 50 mM Tris at pH 8.0 for a minimum of 3 days at room temperature (20 °C).

### Transmission electron cryomicroscopy

A fibril solution (14 µg/mL) was applied onto glow-discharged C-flat holey carbon grids (CF 1.2/1.3–2 C) and after backside blotting, plunged into liquid ethane. Images were collected at 300 kV in two sessions, initially with an FEI Falcon I direct electron detector (DED) mounted on an FEI F30 electron microscope at a total exposure of ~20 e/Å² and later with a K2 Summit (Gatan) DED mounted on an FEI Titan Krios electron microscope. The K2 Summit DED was operated in super-resolution mode to collect movies with 73 frames (0.3 s/frame) and a total exposure of ~40 e/Å^2^.

### Image processing

The movie frames collected on the K2 Summit were background corrected before motion correction was applied by aligning the frames with the program Unblur^37^. All frames were used in the final frame sums. Furthermore, the images collected on the Titan Krios were corrected for magnification distortion using the program mag_distortion_correct^38^. Fibril coordinates were selected using the program TigrisDisplay^39^. Fibrils were grouped according to their crossover length and width. The FREALIX helical image-processing software was used for 3D reconstruction. Axial symmetry of the fibrils was identified by comparison of asymmetric and twofold-symmetrized reconstructions. When both reconstructions gave similar results, a twofold axial symmetry was assumed, otherwise no axial symmetry was assumed (Supplementary Figure 4). Helical symmetry was imposed for all morphologies by using a helical rise calculated from the crossover distances and cross-β repeat (Supplementary Table 1). The polarity of the analyzed fibrils was determined by FREALIX to ensure the correct alignment during the reconstruction.

### MAS solid-state NMR spectroscopy

Packing of the MAS rotor was accomplished by sedimenting 12 mg of fibrils at 21,000 × *g* into the rotor, using a benchtop centrifuge. A 3.2-mm thin-wall ZrO_2_ rotor with vespel cap (CortecNet, Voisins Le Bretonneux, France) was employed with one house-made Teflon spacer (1 mm) at the bottom of the rotor. Proton-driven spin diffusion experiments^40^ with a mixing time of 50 ms were recorded using a 400 MHz Bruker Avance III spectrometer (Bruker BioSpin, Karlsruhe). Sequential assignment was accomplished with 2D versions of NCACX and NCOCX experiments^41^, conducted using a 750-MHz Bruker Avance III spectrometer (Bruker BioSpin, Karlsruhe). Carbon–carbon mixing was achieved by 50 ms of proton-driven spin diffusion. Nonuniform sampling (NUS) was employed to increase the sensitivity of the NCACX and NCOCX experiments^42^. Exponentially decaying sampling densities were used with 50% NUS sparsity and a 3-ms time constant in the ^15^N dimension. Spectra were processed with the NUS plug-in of TopSpin 3.2 (Bruker BioSpin, Karlsruhe), using the compressed sensing option. For all measurements, the temperature of the probe was adjusted to 0 °C. All experiments used 100 kHz of ^1^H decoupling using SPINAL-64^43^ and 10 kHz of MAS spinning. Spectra were analyzed with CcpNmr Analysis 2.4.0^44^. Secondary structure analysis was accomplished with TALOS+^29^ based on the ^13^Cα, ^13^Cβ, ^13^C backbone carbonyl, and ^15^N backbone amide chemical shifts (Supplementary Table 3)^29^. The analyzed fibrils were formed under conditions identical to our cryo-EM samples. They were structurally indistinguishable from those used for reconstruction as judged by negative-staining TEM (Supplementary Figure 8a).

### Theoretical diversity of fibril cross-sections

To generate all possible constellations of fibril cross-sections consisting of 1 ≤ *n* ≤ 12 parallelograms, we used an iterative approach, in which we set out from the fibril consisting of a single parallelogram (*n* = 1), that was deposited within a list *L*_1_. Assuming only in-register LSIs and SSIs, we then added one adjacent parallelogram to the existing cross-section to generate all possible cross-sections containing two parallelograms (*n* = 2). These cross-sections were temporarily stored within a second list *L*_2_ (Supplementary Figure 9a). Nonredundant cross-sections were added to *L*_1_ and the remaining cross-sections in *L*_2_ were deleted. This cycle was repeated, always adding one parallelogram after the other until the value *n* = 12 was reached.

### Data availability

The fibril structural data that support the findings of this study have been deposited and made available in the Electron Microscopy Data Bank. Morphology I was previously made available with the accession no. EMD-3128. The newly generated fibril structures from morphology II to X are also publicly available (accession numbers for morphology II: EMD-3986, III: EMD-3987, IV: EMD-3988, V: EMD-3989, VI: EMD-3990, VII: EMD-3991, VIII: EMD-3992, IX: EMD-3993, and X: EMD-3994). Other data are available from the corresponding authors upon reasonable request.

## Electronic supplementary material

Supplementary Information
