# Supplementary material for: Physical basis of amyloid fibril polymorphism
Source: Nat Commun. 2018 Feb 16;9:699. doi: 10.1038/s41467-018-03164-5 (PMC5816019; doi:10.1038/s41467-018-03164-5)
Supplement: Supplementary file 1 — Supplementary Information [file 41467_2018_3164_MOESM1_ESM.pdf]

Physical basis of amyloid fibril polymorphism

William Close[1†], Matthias Neumann[2†], Andreas Schmidt[1†], Manuel Hora[3,4],

Karthikeyan Annamalai[1], Matthias Schmidt[1], Bernd Reif[3,4],

Volker Schmidt[2], Nikolaus Grigorieff[5*], Marcus Fändrich[1*]

Supplementary Figures

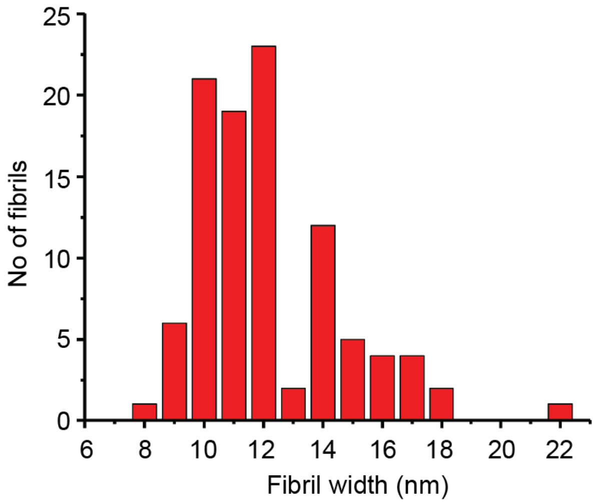

Supplementary Figure 1.

Variation of the width of AL1 peptide fibrils.

Analysis of 100 fibrils using cryo-EM micrographs.

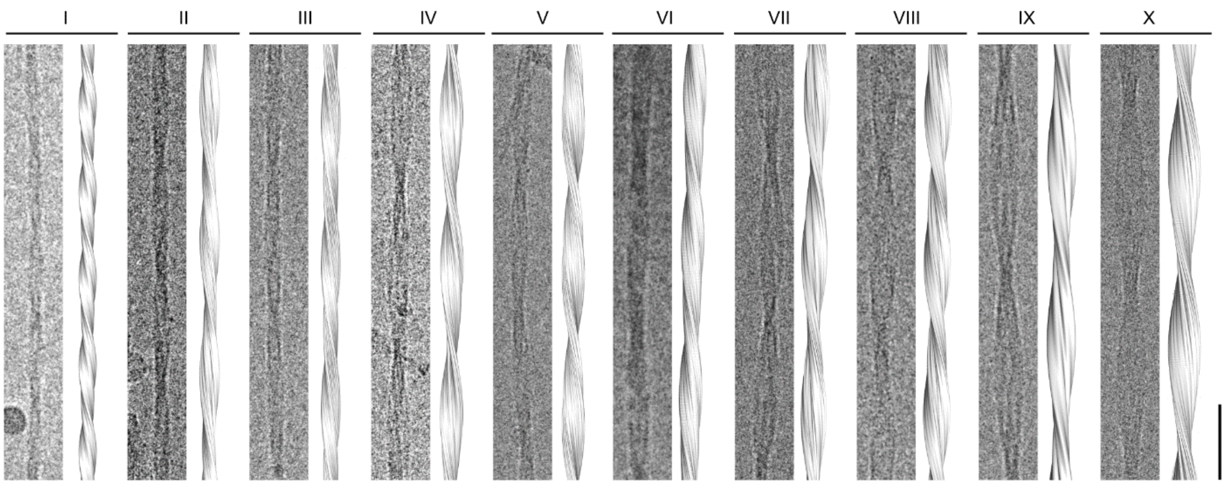

Supplementary Figure 2.

Side by side comparison of reconstructions and raw images. Pairing of example fibrils from cryo-EM micrographs with their three-dimensional reconstructions for morphologies I to X. Reconstructions of morphologies I-V were filtered to 10 Å and morphologies VI-X to their FSC values at 0.143. The data from morphology I were previously published[1] (Scale bar, 50 nm).

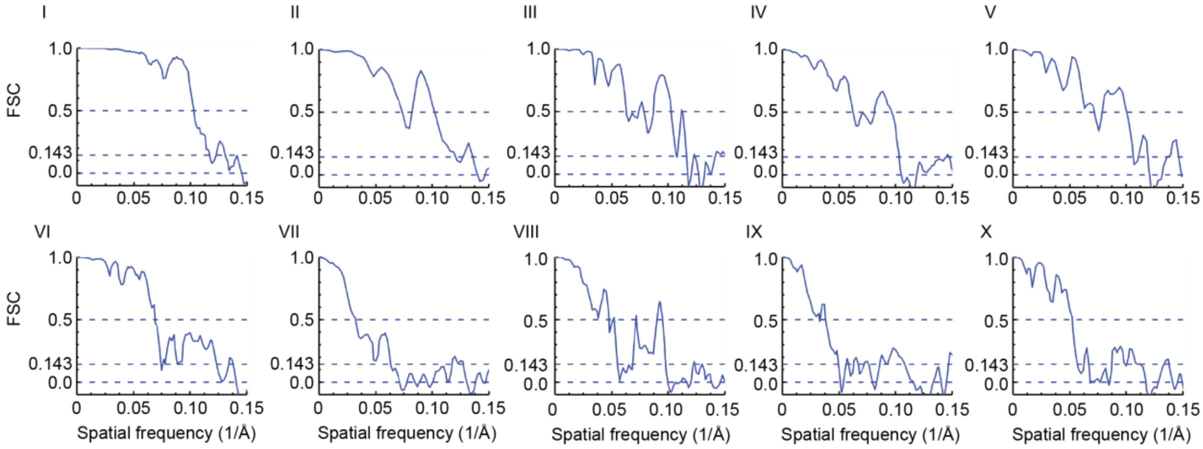

Supplementary Figure 3.

FSC curves of fibril morphologies I-X.

The values of the resolution according the threshold of the FSC curves at 0.5 and 0.143 (dashed lines) are given in Table S1. The data from morphology I were taken from a previous study[1] and are plotted here for comparison.

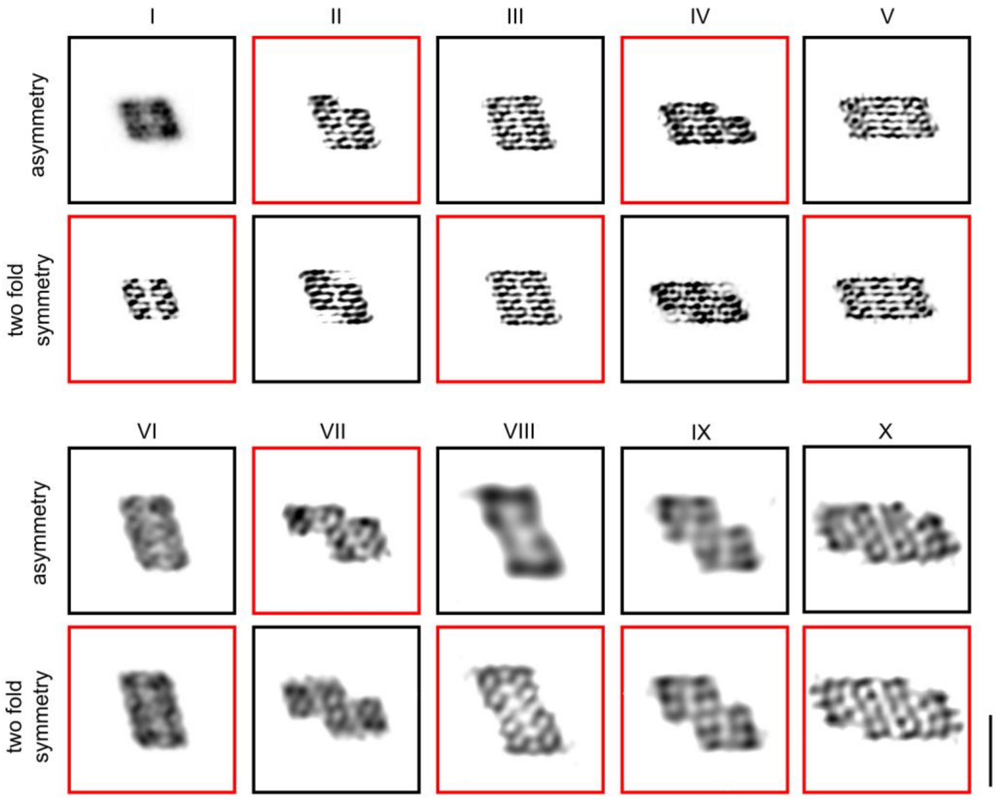

Supplementary Figure 4.

Reconstructed fibril cross-sections testing different symmetries. Cross-sectional views of the AL1 peptide fibril morphologies testing no axial symmetry and twofold axial symmetry. Red box: symmetry used in the final analysis. The data from morphology I were previously published[1]. Reconstructions of morphologies I-V were filtered to 10 Å and morphologies VI-X to their FSC values at 0.143 (Scale bar, 10 nm).

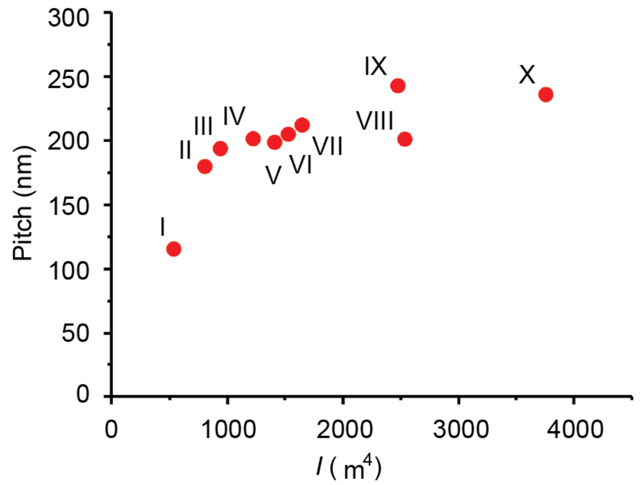

Supplementary Figure 5.

Correlation of *I* and fibril pitch.

The values of *I* were determined as described in the Method section. The pitch value was taken from supplementary table 1.

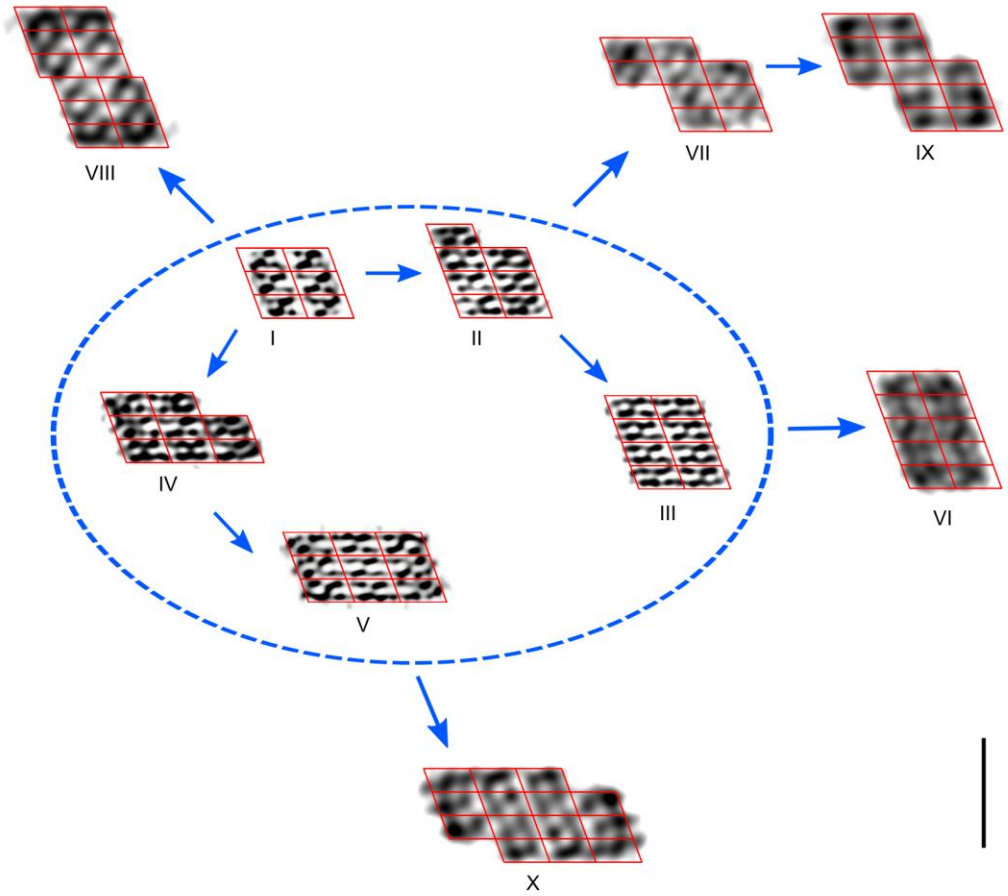

Supplementary Figure 6.

Morphological family tree of AL1 peptide fibrils.

Dashed blue eclipse highlights morphologies I to V which were reconstructed to resolutions of 1.0 nm or better. The surrounding morphologies VI to X were reconstructed at resolutions of 1.3 to 2.0 nm. All fibril cross-sections were superimposed with an arrangement of a parallelograms (red). Arrows connect morphologies that differ by the smallest number of parallelograms that have to be added without the requirement of parallelograms to be removed. This figure is not meant to imply a kinetic assembly pathway. Reconstructions of morphologies I-V were filtered to 10 Å and morphologies VI-X to their FSC values at 0.143. The data from morphology I were previously published[1] (Scale bar, 10 nm).

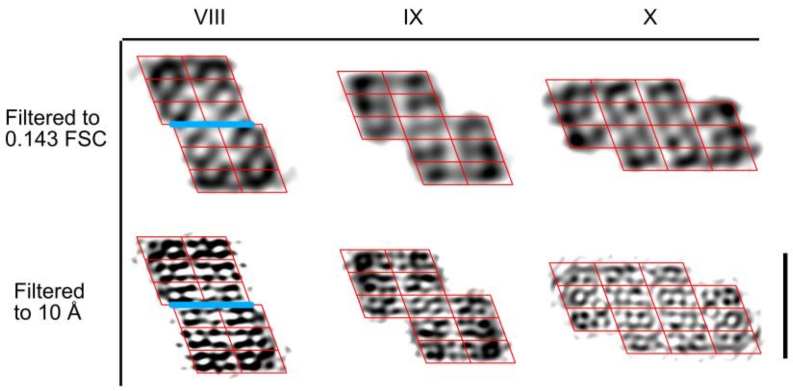

Supplementary Figure 7.

Evidence for elongated density cores in fibril morphologies VIII, IX and X.

Top row: fibril reconstructions filtered to the 0.143 FSC value of the reconstructions (VIII: 18 Å, IX: 20 Å, X: 18 Å). Bottom row: fibril reconstructions filtered to 10 Å suggesting the presence of elongated regions spaced at approximately 11 Å. All reconstructions were based on a single cryo-EM fibril image. Cross-sectional views were superimposed by a lattice of parallelograms (red). The light blue line in morphology VIII indicates an off-register block interface that is different from the canonical in-register one (Scale bar, 10 nm).

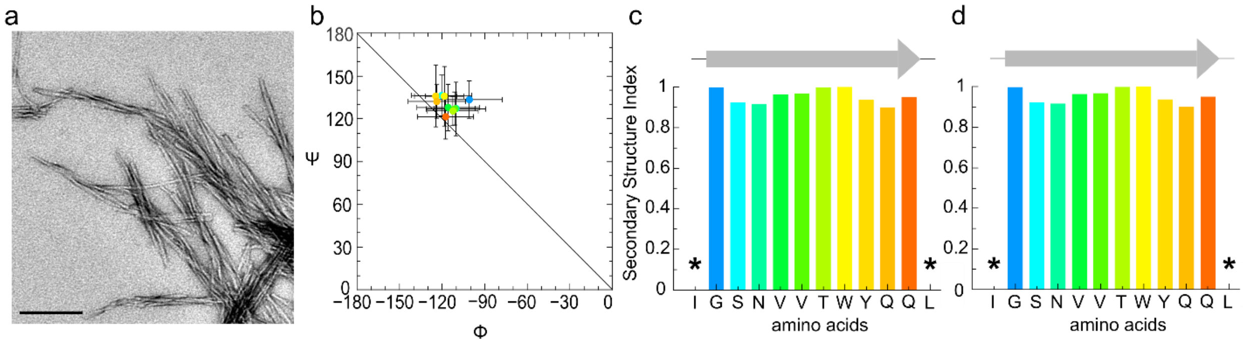

Supplementary Figure 8.

Peptide conformation in the ss-NMR sample.

a Negative stain TEM image of the sample analyzed by ss-NMR (Scale bar, 200 nm). **b** Upper left quadrant of the Ramachandran plot with Φ/Ψ pairs of the AL1 peptide as obtained by TALOS+[2]. Error bars represent standard deviations. **c,d** The β-sheet probability calculated with the Secondary Structure Index for each amino acid by the program TALOS+[2]. The two plots implement the two sets of chemical shifts obtained for residues Ser3 and Thr7. Asterisk: no Φ/Ψ pairs and secondary structure could be assigned to the first and last amino acid. The same color code was used for the amino acids in panels b to d.

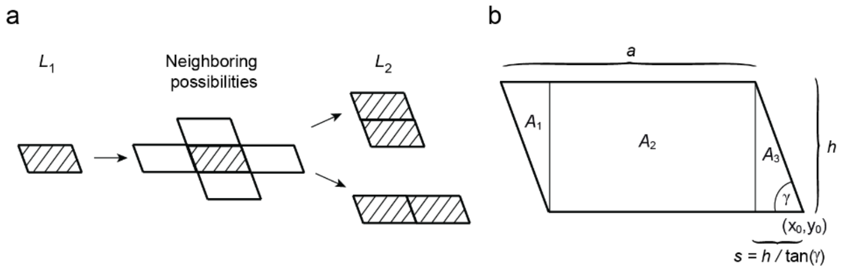

Supplementary Figure 9.

Computational generation of cross-sectional arrangements and calculation of *I*(*P*).

a A fibril cross-section consisting of a single parallelogram (n = 1) belonging to L_1 (hashed). Addition of neighbouring parallelograms in all possible positions (unfilled) and removal of redundant configurations yields two remaining cross-sections with n = 2 in L_2. **b** Calculation of $I(P)$ was achieved by computing I of the three areas labelled A_1, A_2 and A_3 as described by Equations (5)-(7).

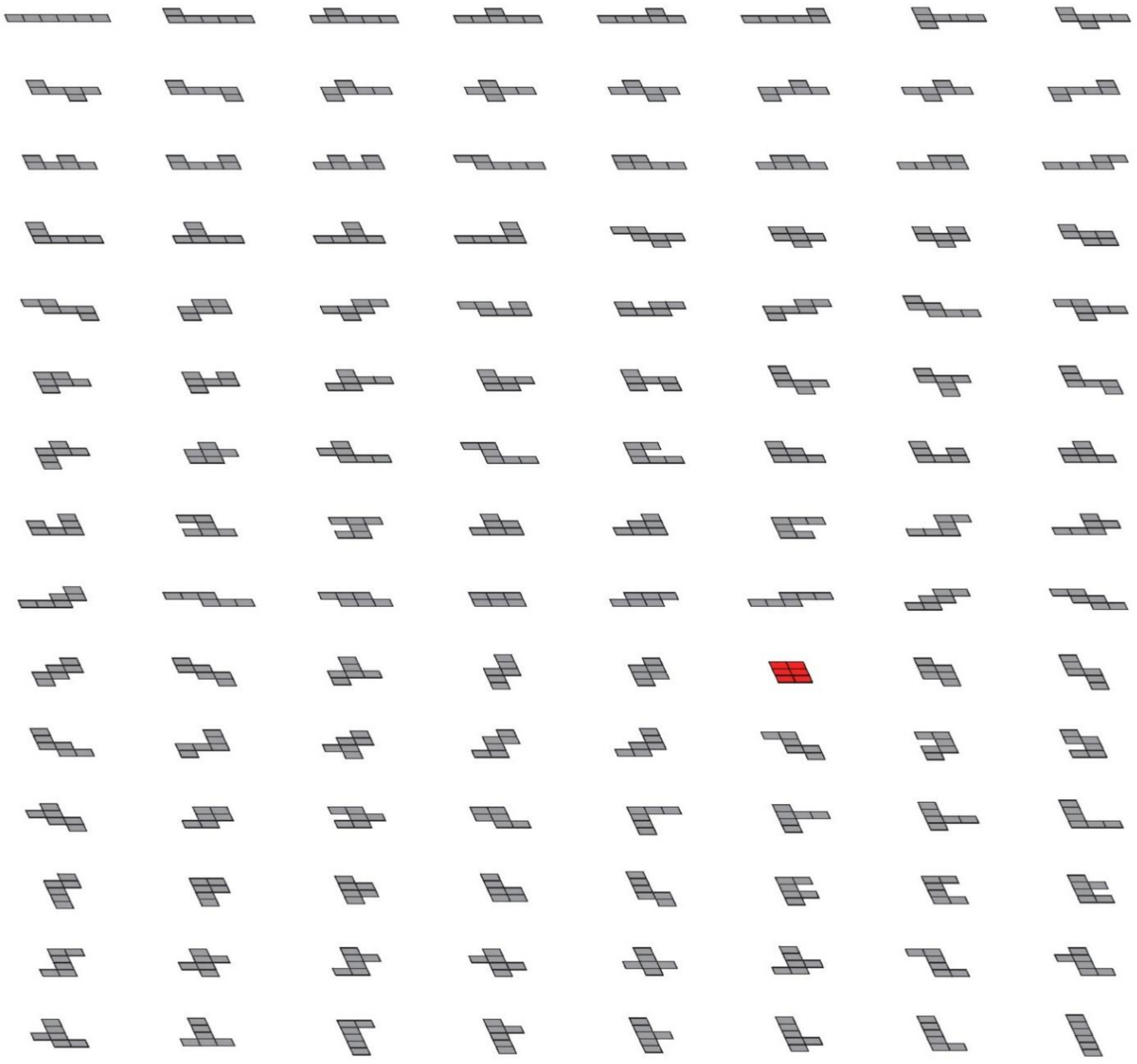

Supplementary Figure 10.

Theoretically possible fibril morphologies consisting of six PFs.

All images refer to the structure of the fibril cross-section, represented here as an arrangement of building blocks (parallelograms). The experimentally observed fibril morphology I is highlighted in red.

a

n	pA
1	1
2	2
3	4
4	13
5	35
6	120
7	392
8	1,405
9	4,998
10	18,378
11	67,792
12	253,509
Total	346,649

b

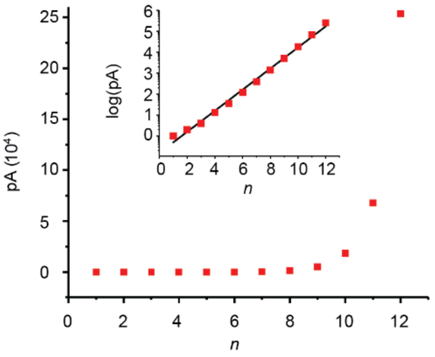

Supplementary Figure 11.

Parallelogram arrangement possibilities. a Number of possible arrangements (pA) of building blocks for a given number pf PFs *n*. **b** Plot of the data from panel a. Insert shows a logarithmic y-scale. These data were fitted with the formula log(pA) = 0.5*n* -0.8 (R = 0.99).

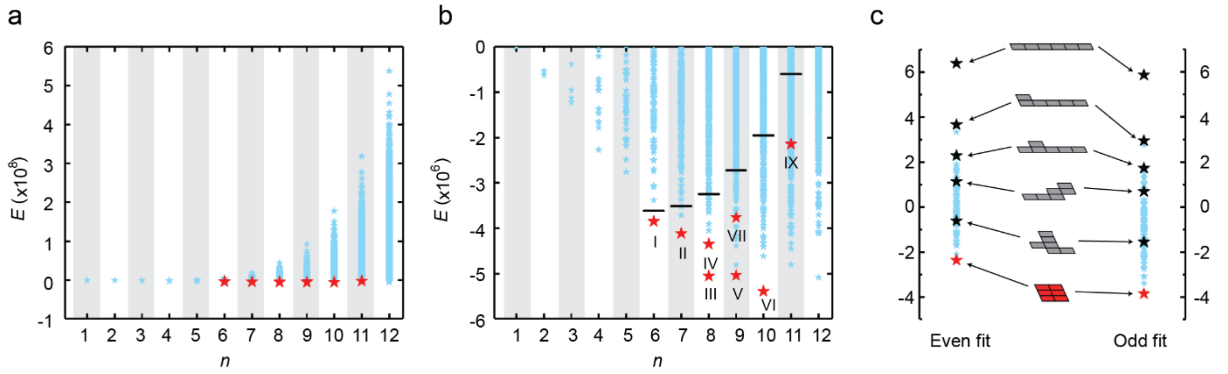

Supplementary Figure 12.

Mathematical analysis of the observed and theorized fibril morphologies.

a Plot of *E* for all theorized fibril morphologies or cross-sections (blue) versus *n* as obtained by the odd fit (grey columns). Hence, only white columns have predictive power for the odd fit. Red symbols: experimentally observed fibril morphologies. **b** Close-up from panel a. Black horizontal division markers show the 1% cutoff of the most stable morphologies for each *n* value. **c** Plot of *E* of all fibril morphologies consisting of $n = 6$ PFs as derived from odd and even fit. Vertical arrangement of parallelograms show different morphologies ranging across the spectrum of *E* values represented by black stars.

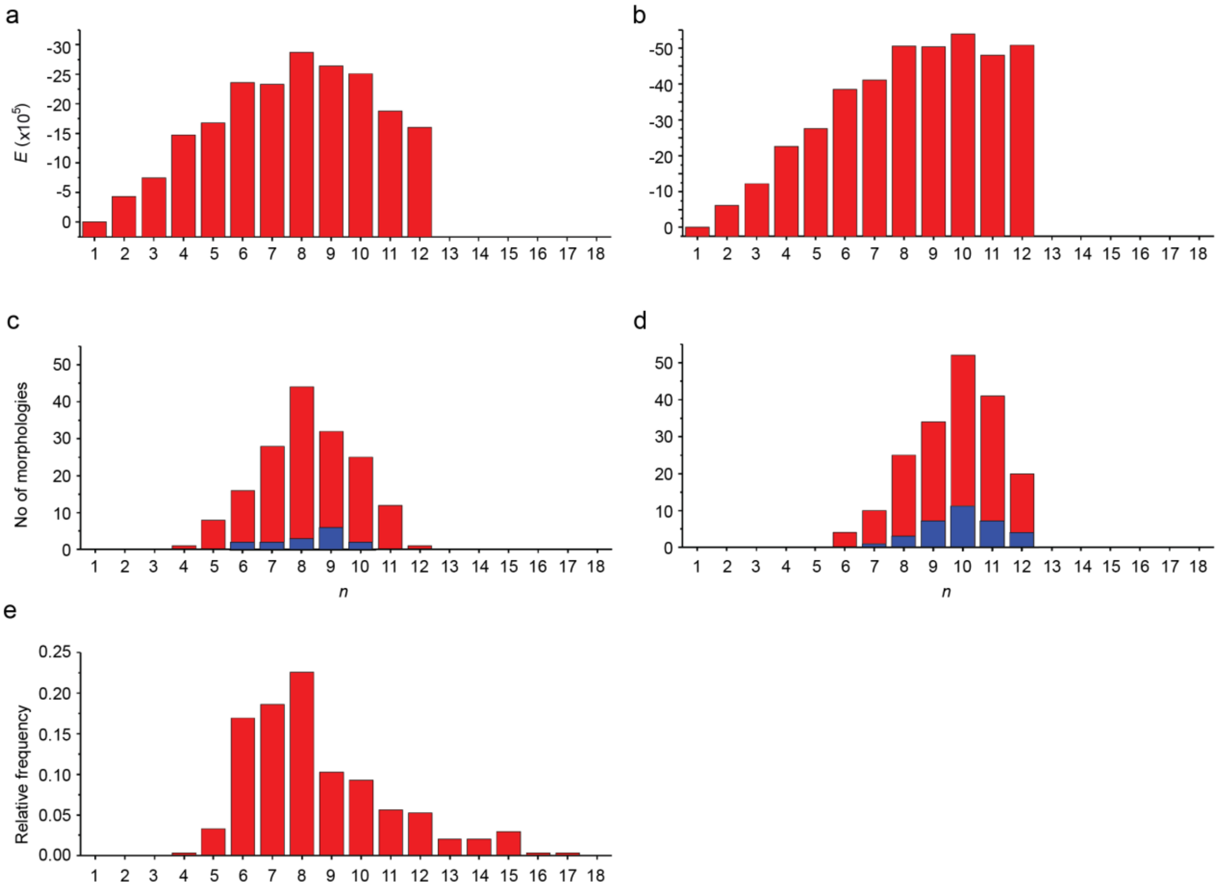

Supplementary Figure 13.

Definition of an intermediate region of *n* with highly stable fibrils.

a-b Lowest *E* values using the even and odd fit respectively. **c** The number of morphologies under the energy threshold -1x10^6 (red) -2x10^6 (blue) for the even fit data. **d** The number of morphologies under an *E* threshold -3x10^6 (red) -4x10^6 (blue) for the odd fit data. **e** Frequency of fibrils displayed in terms of *n* values based on MPL measurements. The MPL data were taken from a previous study[1].

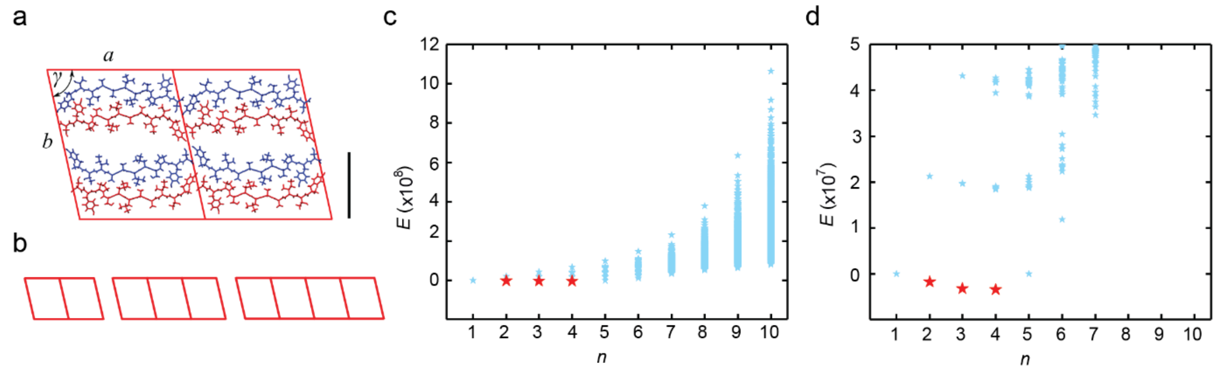

Supplementary Figure 14.

Mathematical analysis of TTR fibrils showing observed and theorized morphologies.

a Structural representation of a two-block fibril morphology obtained from TTR(105-115) peptide by cryo-EM as previously published[11]. Two building blocks are packed side by side. The fitting of the parallelogram boundaries resulted in approximate values of a = 3.799 nm, b = 4.561 nm and γ = 77.66˚ (Scale bar, 2 nm). **b** Building block arrangements for the observed fibrils. **c** Plot of E for all theorized fibril morphologies or cross-sections that were obtained for fibrils with $1 \leq n \leq 10$ and assuming only in-register LSIs and SSIs (blue) versus n. Red symbols: The three observed fibril morphologies as published[11] present the lowest E values of all theorized configurations. **d** Close-up from panel c. In the fit, we used the additional constraint that $E_l^{\,2} + E_s^{\,2}$ is bounded by the upper constant 10^{16} based on the results of the fit in Supplementary Table 4. In this way, we avoid the possibility that the cost function becomes infinitely small for arbitrarily large values but allows for a certain degree of freedom for fitting. The fibrils were not subdivided into even or odd PF numbers and Equation (1) was simplified by omitting the E_e term. The obtained values for E_s and E_l were -18.1 x10^5 and 212.1 x10^5 a.u., respectively. For consistency with our analysis of AL1 peptide fibrils, E_l refers to interactions across edge a, whereas E_s refers to interactions across edge b. These data demonstrate that our theory is also able to rationalize the three observed TTR(105-115) peptide fibril morphologies.

Supplementary Tables
Supplementary Table 1.

Structural statistics of the analyzed ten different fibril morphologies

	**Morphology**									
	**I**	**II**	**III**	**IV**	**V**	**VI**	**VII**	**VIII**	**IX**	**X**
**Pitch, nm**	115.4	179.8	193.8	201.5	198.7	205	212.2	201.2	242.6	235.8
**Fibril width, nm**	8.89	13.49	13.26	15.54	15.75	14.54	15.97	16.7	17.12	18.71
**Pixel size on the specimen, Å**	2.11	2.1	2.1	2.1	2.1	2.11	2.1	2.1	1.05	1.05
**Resolution (FSC at 0.5), Å**	9.8	13.9	15.9	16.1	13.9	14.5	31	21.1	30.4	19.1
**Resolution (FSC at 0.143), Å**	8.3	8.4	9.3	9.7	9.4	13.4	15.9	18.2	20.3	17.7
**Total length of non-overlapping segments, nm**	13,361	3,753	6,290	2,164	1,673	1,092	665	360	356	374
**Number of fibrils**	11	10	9	6	3	5	2	1	1	1
**Total number of contributing subunits**	28,488	8,003	13,412	4,615	3,568	30,376	852	1,146	882	613
**Scoring function step size, pixel**	10-12.8	10	10	10	10	10	10-14	10	21	10
**Size of reconstruction, nm**	60.77	31.08	60.06	64.26	38.22	56.08	60.07	60.05	60.07	60.07
**Repeat distance, nm**	0.469	0.469	0.469	0.469	0.469	0.469	0.469	0.469	0.469	0.469
**Size of segments, nm**	5.2-6.7	4.09	5.77	4.17	3.3	8.2	8.3-11.9	3.3	17.3	8.3
**Rotation per subunit, °**	1.46	0.94	0.88	0.85	0.84	0.82	0.8	0.84	0.7	0.72

Various reconstruction data resulting in the ten different fibril morphologies. The data from morphology I were taken from a previous publication[1].

Supplementary Table 2.

Dimensions of the parallelogram as determined by a cross-correlation analysis of fibril morphologies I to V

Morphology	Length of axis *a* (nm)	Length of axis *b* (nm)	Value of angle *γ* (°)
**I**	4.35 ± 0.11	2.39 ± 0.11	67 ± 0.4
**II**	4.10 ± 0.03	2.23 ± 0.06	71.6 ± 0.8
**III**	4.33 ± 0.04	2.21 ± 0.07	73.7 ± 0.1
**IV**	4.04 ± 0.09	2.36 ± 0.03	66.2 ± 1.8
**V**	4.26 ± 0.01	2.27 ± 0.07	70.4 ± 0.4
**Average**	4.22 ± 0.31	2.29 ± 0.09	69.8 ± 3.2

The data from morphology I were taken from a previous publication[1].

Supplementary Table 3.

Nuclear magnetic resonance chemical shifts

No.	residue	atom	chem. shift (ppm)	No.	residue	atom	chem. shift (ppm)
1	Ile	C	172.2	8	Trp	CB	32.1
1	Ile	CA	59.3	8	Trp	CD1	126.8
1	Ile	CB	37.2	8	Trp	CD2	131.1
1	Ile	CD1	11.8	8	Trp	CE2	135.8
1	Ile	CG1	26.3	8	Trp	CE3	119.5
1	Ile	CG2	14.9	8	Trp	CG	111.3
2	Gly	C	171.1	8	Trp	CH2	122.5
2	Gly	CA	42.6	8	Trp	CZ3	120.9
2	Gly	N	115.9	8	Trp	N	129.7
3	Ser	C	170.0	9	Tyr	C	171.2
3 *	Ser	CA	54.4	9	Tyr	CA	54.2
3 *	Ser	CA	55.3	9	Tyr	CB	41.2
3 *	Ser	CB	63.6	9	Tyr	CD1	131.2
3 *	Ser	CB	64.5	9	Tyr	CD2	131.2
3	Ser	N	120.6	9	Tyr	CE1	115.6
4	Asn	C	172.8	9	Tyr	CE2	115.6
4	Asn	CA	50.3	9	Tyr	CG	127.2
4	Asn	CB	40.0	9	Tyr	CZ	154.6
4	Asn	N	122.3	9	Tyr	N	121.4
4	Asn	ND2	111.8	10	Gln	CA	51.3
5	Val	C	172.9	10	Gln	CD	177.6
5	Val	CA	58.5	10	Gln	CG	32.4
5	Val	CB	33.8	10	Gln	NE2	110.7
5	Val	CG1	19.8	11	Gln	C	171.1
5	Val	N	125.5	11	Gln	CA	51.3
7	Thr	C	170.6	11	Gln	CB	30.2
7 *	Thr	CA	59.7	11	Gln	CD	177.7
7 *	Thr	CA	59.0	11	Gln	N	126.5
7 *	Thr	CB	66.7	11	Gln	NE2	105.7
7 *	Thr	CB	67.5	12	Leu	C	179.9
7 *	Thr	CG2	21.3	12	Leu	CA	54.0
7 *	Thr	CG2	19.1	12	Leu	CB	41.4
7	Thr	N	125.2	12	Leu	CD1	23.4
8	Trp	C	171.6	12	Leu	CG	25.9
8	Trp	CA	54.6	12	Leu	N	132.6

* The observation of two sets of resonances at these two residues indicates different chemical environments. This finding is consistent with our model which places the odd numbered amino acids to the outside of the dimer. Different environments arise depending on whether the dimer surface is solvent exposed or packed against another dimer.

Supplementary Table 4.

Values of E_s, E_l and E_e as obtained by the fit

	**Odd *n* values used for fitting**	**Even n values used for fitting**
**E_s, [x10^4 a.u.]**	- 54.7	- 44.2
**E_l, [x10^4 a.u.]**	- 62.4	- 33.1
**E_e, [x10^4 a.u.]**	75.7	60.3

All values are given in arbitrary units (a.u.).

Supplementary Methods

Cross-correlation analysis of the fibril cross-sections

Cross-sections representing the reconstructed density maps of morphologies II-X were extracted at 6.3 Å thick slices, sharpened and filtered at an FSC value of 0.143 or a minimum of 10 Å using the program bfactor[3] and averaged using EMAN2[4]. The cross-sections were analyzed to search for repetitive structural units leading to the lattice constants visible in the fibril cross-sections as previously described for morphology I[1]. The autocorrelation function was calculated using the program Spider[5] to determine these lattice vectors as the distances between correlation maxima.

Measurement of I, n_l, n_s, and n_e

The characteristics n_l and n_s were determined by counting all the adjoining parallelograms within a fibril cross-section (Supplementary Figure 9a). The number of empty sharp corners n_e was defined by first generating an L shaped template with three adjoining parallelograms and a missing parallelogram in one of the sharp corners. This template was then overlaid in various positions over a particular cross-section to determine a positive or negative correlation for each parallelogram. When all four positions of the template matched the analyzed cross-section, it was counted as one n_e. Once the entire cross-section had been overlaid, the template was then rotated 180˚ and the process was repeated.

To compute *I* from a fibril cross-section, the cross-section was translated in the *x*-*y* plane such that its barycenter coincided with the origin. This centred cross-section, denoted here by S', was used to compute *I* as defined by Equation (2).

$$I(S') = \int_{S'} x^2 + y^2 \ d(x,y) \tag{2}$$

To speed up the mathematical process, we analytically computed the integral $I(S')$ rather than obtaining it by numerical integration. That is, we obtained $I(S')$ from the sum of the polar moments of inertia $I(P)$ of the individual parallelograms $P_1, \ldots, P_n$, as outlined by Equation (3).

$$I(S') = I(P_1) + \cdots + I(P_n) \tag{3}$$

To compute $I(P)$, we divided each parallelogram into 3 parts A_1, A_2 and A_3 as shown in Fig. 9b and obtained $I(P)$ as the sum of the polar moments of inertias of the parts A_1, A_2 and A_3 as described by Equation (4).

$$I(P) = I(A_1) + I(A_2) + I(A_3) \tag{4}$$

$I(A_1)$, $I(A_2)$ and $I(A_3)$ were calculated as described by Equations (5)-(7).

$$I(A_1) = \int_{A_1} x^2 + y^2 \,\mathrm{d}(x, y) = \frac{6h^2(x_0^2 + y_0^2) + 4y_0h^3 + h^4}{12\tan(\gamma)} + \frac{2x_0h^3}{3\tan^2(\gamma)} + \frac{h^4}{4\tan^3(\gamma)} \tag{5}$$

$$\begin{aligned} I(A_2) &= \int_{A_2} x^2 + y^2 \,\mathrm{d}(x, y) \\ &= \frac{h}{3}\left((x_0 + a)^3 - \left(x_0 + \frac{h}{\tan(\gamma)}\right)^3\right) + \frac{a - h/\tan(\gamma)}{3}\left((y_0 + h)^3 - y_0\right) \end{aligned} \tag{6}$$

$$I(A_3) = \int_{A_3} x^2 + y^2 \, \mathrm{d}(x,y) = \frac{6h^2((x_0 + a)^2 + y_0^2) + 8y_0h^3 + 3h^4}{12\tan(\gamma)} + \frac{h^3(x_0 + a)}{3\tan^2(\gamma)} + \frac{h^4}{12\tan^3(\gamma)} \quad (7)$$

In Equations (5) - (7) the base length of the parallelogram is denoted by a, the height by h and the angle in the corner by γ. The lowest right hand corner of the parallelogram was used as a coordinate reference point for the determination of I as indicated by (x_0,y_0).

Global fit of the data

Having obtained the characteristics n_l, n_s, n_e and I from all 346,649 fibril morphologies we split the resulting data set into two parts, one containing all fibril morphologies with even n values and the other containing all fibrils with odd n values. Each part of the data set was then subjected to a fit of all its fibril morphologies. In this fit we adjusted the values of E_l, E_s and E_e such that the cost-function (8)

$$\max\{\, E(S): S \in S_o\} - \min\{E(S): S \in S_{no}\} \quad (8)$$

was minimized by simulated annealing[6]. In this cost function, $E(S)$ denotes the energy value of a given fibril cross-section S while the set of experimentally observed cross-sections is referred to as S_o. The set of cross-sections that were not observed by cryo-EM is referred to as S_{no}. The optimization toolbox of Matlab[7] was used to perform simulated annealing. The factor α within Equation (1) was not determined during fitting but was set to 1.

Supplementary References

1. Schmidt, A., Annamalai, K., Schmidt, M., Grigorieff, N. & Fändrich, M. Cryo-EM reveals the steric zipper structure of a light chain-derived amyloid fibril *Proc. Natl. Acad. Sci. U.S.A.* **113**(22), 6200-6205 (2016).
2. Shen, Y., Delaglio, F., Cornilescu, G. & Bax, A. TALOS+: A hybrid method for predicting protein backbone torsion angles from NMR chemical shifts *J. Biomol. NMR.* **44**(4), 213-223 (2009).
3. Grigorieff, N. bfactor, Version 1.04. Grigorieff lab, Janelia Research Campus, Virginia, United States (2012). Available at grigoriefflab.janelia.org/ bfactor. Accessed May 2016.
4. Tang, G. *et al.* EMAN2: An extensible image processing suite for electron microscopy *J. Struct. Biol.* **157**(1), 38-46 (2007).
5. Frank, J. *et al.* SPIDER and WEB: Processing and visualization of images in 3D electron microscopy and related fields *J. Struct. Biol.* **116**, 190–199 (1996).
6. Kroese, D. P., Taimre, T. & Botev, Z. I. Handbook of Monte Carlo Methods. *John Wiley & Sons*, New York, (2011).
7. MATLAB 2015b, The MathWorks, Inc., Natick, Massachusetts, United States (2015).
